# Therapeutic Potential of Melatonin Counteracting Chemotherapy-Induced Toxicity in Breast Cancer Patients: A Systematic Review

**DOI:** 10.3390/pharmaceutics15061616

**Published:** 2023-05-30

**Authors:** Eva Ramos, Javier Egea, Francisco López-Muñoz, Emilio Gil-Martín, Alejandro Romero

**Affiliations:** 1 Department of Pharmacology and Toxicology, Faculty of Veterinary Medicine, Complutense University of Madrid, 28040 Madrid, Spain; 2 Molecular Neuroinflammation and Neuronal Plasticity Research Laboratory, Hospital Universitario Santa Cristina, Instituto de Investigación Sanitaria-Hospital Universitario de la Princesa, 28006 Madrid, Spain; 3 Institute Teófilo Hernando for Drug Discovery, Department of Pharmacology, School of Medicine, Autonomous University of Madrid, 28029 Madrid, Spain; 4 Faculty of Health, Camilo José Cela University of Madrid (UCJC), 28692 Madrid, Spain; 5 Neuropsychopharmacology Unit, Hospital 12 de Octubre Research Institute, 28041 Madrid, Spain; 6 Department of Biochemistry, Genetics and Immunology, Faculty of Biology, University of Vigo, 36310 Vigo, Spain

**Keywords:** breast cancer, melatonin, chemotherapy, toxicity, adjuvant therapy

## Abstract

The purpose of this systematic review is to provide an overview of the existing knowledge on the therapeutic potential of melatonin to counteract the undesirable effects of chemotherapy in breast cancer patients. To this aim, we summarized and critically reviewed preclinical- and clinical-related evidence according to the PRISMA guidelines. Additionally, we developed an extrapolation of melatonin doses in animal studies to the human equivalent doses (HEDs) for randomized clinical trials (RCTs) with breast cancer patients. For the revision, 341 primary records were screened, which were reduced to 8 selected RCTs that met the inclusion criteria. We assembled the evidence drawn from these studies by analyzing the remaining gaps and treatment efficacy and suggested future translational research and clinical trials. Overall, the selected RCTs allow us to conclude that melatonin combined with standard chemotherapy lines would derive, at least, a better quality of life for breast cancer patients. Moreover, regular doses of 20 mg/day seemed to increase partial response and 1-year survival rates. Accordingly, this systematic review leads us to draw attention to the need for more RCTs to provide a comprehensive view of the promising actions of melatonin in breast cancer and, given the safety profile of this molecule, adequate translational doses should be established in further RCTs.

## 1. Introduction

Cancer is one of the most prevalent diseases and one of the leading causes of death worldwide [1], accounting for ~10 million deaths each year, which alarmingly continues to rapidly increase worldwide. The International Agency for Research on Cancer (IARC) has recently estimated a worsening of the global burden of the disease, from 14.1 million new cases (in 2012) to 21.7 million expected by 2030 [2]. In this same sense, it is expected that up to 40% of the adult population of men and women will be diagnosed with cancer throughout their lives, especially in developed countries. In the case of breast cancer, one of the reasons argued to explain the increasing trend in prevalence rates is industrialization, particularly artificial light at night (ALAN) [3]. Indeed, several studies have reported that prolonged and repeated exposure to ALAN [4] or sleep disturbance women with rotating night shifts [5] are key risk factors for this disease. Indeed, breast cancer is the most common cancer in women worldwide, accounting for ~25% of all cancer cases and affecting more than 2.2 million women each year [6]. Moreover, it is the second leading cause of cancer death in women (1 in 6 cancer deaths), exceeded only by lung cancer. However, despite the increase in overall cancer incidence, hopefully, the mortality rate of some tumors is being contained, which can be attributed to the immense research effort of the last decades that has allowed promising advances in immunotherapy by harnessing innate and/or adaptive immunity and precision medicine [7]. In this regard, breast cancer is a paradigmatic example of a highly prevalent tumor, whose in-deep study has achieved significant improvements in molecular pathogenesis, biomarkers to reach an early diagnosis and better outline the prognosis of patients, and therapeutic targets that improve the efficacy of clinical management. Additionally, the extension of prediagnostic screenings among the general population and/or risk cohorts has made it possible to routinely detect early tumors or even identify asymptomatic cases.

The multiplicity of treatments currently available to reduce disease severity and increase total and disease-free survival, including surgery, radiation therapy, chemotherapy, hormone therapy, targeted therapy, and immunotherapy, has been supplemented by multimodal combined therapies, which preserve therapeutic efficacy and patient life expectancy while reducing undesirable side-effects in diagnosed patients [8]. Despite this, the outcome of the patients, especially for metastatic breast cancer, is not satisfactory due to high recurrence and low survival rates [9]. Furthermore, some of the conventional treatment modalities are associated with non-specific toxicity responsible for detrimental side effects that have a significant impact on the quality of life of patients [10]. In this context, adjuvant radio/chemotherapy dosage regimens that achieve maximum drug efficacy confer a therapeutic advantage, although they may also result in off-target toxicity. Thus, thrombo and lymphocytopenia and other undesirable hematological and immunological disorders, together with fatigue, insomnia, cognitive impairment, peripheral neuropathy, depression, and psychological stress, are some of the adverse side effects of conventional chemotherapy lines in breast cancer [11]. Therefore, the development of effective practices in clinical interventions and a complementary adjunctive defense against toxicity associated with breast cancer therapy is urgent.

Increasing evidence suggests that melatonin (*N*-acetyl-5-methoxytryptamine) is a cell protector endowed with systemic effects against many types of cancer [12,13,14,15,16], including breast cancer [17,18,19,20]. In this regard, melatonin possesses antioxidant properties, which may help to protect cells from damage caused by free radicals and other harmful substances that can contribute to cancer development [21]. Specifically, melatonin directly scavenges free radicals, such as hydroxyl radical, peroxynitrite anion, singlet oxygen, and superoxide anion, thus protecting cells from oxidative stress [22]. Remarkably, the elimination of free radicals non-enzymatically transforms melatonin into metabolites with greater antioxidant capacity, which enabling the removal of 10 reactive species per molecule [23]. Antioxidative protection is especially relevant in the mitochondria of the extrapineal tissues, where more than 90% of body melatonin is synthesized and which, in addition to protecting organelles from reactive species, facilitates energy production [24]. In particular, melatonin activates transcription factors that control the expression of mitochondrial genes, such as complex I and complex IV, as well as influences ATP production and energy metabolism [21]. Furthermore, melatonin upregulates various antioxidant enzymes, such as superoxide dismutase, catalase, and glutathione peroxidase [22], which are responsible for neutralizing free radicals and preventing oxidative insults. Overall, the direct and indirect antioxidant activity of melatonin works together to ensure cell homeostasis and thereby promote health and well-being. Accordingly, several studies have found that individuals with higher melatonin levels show a lower risk of developing breast cancer, and melatonin supplementation may help inhibit the growth and spread of breast cancer cells [25,26]. Given the promising therapeutic potential of melatonin for breast cancer patients, there is great interest in conducting carefully designed multicenter clinical trials enrolling large prospective cohorts to fully understand its potential benefits and risks.

Further, two membrane-associated G protein-coupled melatonin receptors (named MT1 and MT2) are widely expressed in various tissues and organs, including the breast [27]. Some investigations have found that the activation of melatonin receptors in breast cancer cells may have antiproliferative and pro-apoptotic effects, leading to growth inhibition [28,29] and control of tumor angiogenesis [30]. In this respect, it has been reported that melatonin inhibits the development of breast cancer via the MT1 receptor, with an inverse correlation between the expression of the MT1 protein and the degree of malignancy of the tumors [31]. This fact reveals that MT1 may be used as a pathologic biomarker to evaluate breast carcinoma tissue [32]. On the other hand, estrogen has a significant role in the development and progression of breast cancer. Thus, estrogen/progesterone receptor-positive breast cancers include the most common types of breast cancer (75% of all cases). In this sense, melatonin is able to modulate the estrogen/ER signal transduction pathway, displaying an antiestrogenic effect and, therefore, inhibiting the growth of human breast tumors [32,33].

Melatonin has been shown to have anti-inflammatory properties by regulating several immune mechanisms that (i) enhance natural killer cell activity, (ii) inhibit the production of pro-inflammatory cytokines such as interleukin-1 beta (IL-1β), interleukin-6 (IL-6), and tumor necrosis factor-alpha (TNF-α) [34,35], and (iii) protect hematopoietic progenitor cells from radiation therapy and chemotherapy [36]. Likewise, melatonin can inhibit the activation of nuclear factor-kappa B (NF-κB), which reduces the expression of pro-inflammatory genes and helps dampen the inflammatory response [37]. Consequently, the important melatonin-driven modulation of immune and inflammatory responses may be useful for controlling tumor progression and the clinical management of breast cancer patients [38]. Indeed, melatonin has aroused increasing expectations in recent years as an adjuvant in breast cancer therapy, reducing side effects and enhancing the efficacy of chemotherapy [39,40,41]. However, despite promising results from preclinical studies, the use of melatonin as an adjuvant in the treatment of breast cancer is still in the early stages of study, with some results suggesting potential benefits [42] and others not finding any effects [43]. Factors that may contribute to the variability in preliminary clinical results include differences in patient cohorts, dosing regimens, or study design. Therefore, further research to determine optimal dosing protocols and additional randomized clinical studies are necessary.

## 2. Materials and Methods

Systematic reviews and meta-analyses are accurate and reliable tools of evidence-based practice that are becoming the gold standard for collecting evidence related to any topic of interest and facilitating updating, the critical synthesis of information from sometimes confusing and contradictory literature, and optimizing the decision-making by professionals, avoiding biases and misunderstandings. In this regard, we performed a selection and bibliographic analysis according to the systematic review methodology of the PRISMA statement [44]. The review protocol was designed and validated by all authors. Specifically, following the PRISMA guidelines, we compiled the initial studies, assessed the quality and relationship of each one with the topic of interest, extracted their relevant data, and then carried out a critical synthesis. This method was selected to collect all the primary studies that may contain relevant RCTs regarding melatonin co-treatment in breast cancer up to 26 December 2022. We chose the PRISMA protocol to ensure the transparency and reproducibility of the criteria used for the selection and processing of the literature. Below, we describe the step-by-step PRISMA methodology.

### 2.1. Search Strategy

The systematic search for the primary reports was carried out in the main repositories of biomedical information, including Medline (https://pubmed.ncbi.nlm.nih.gov (accessed on 26 December 2022)), Scopus (https://www.scopus.com (accessed on 26 December 2022)), Web of Science (https://www.webofscience.com/wos/woscc/basic-search (accessed on 26 December 2022)), and the Cochrane library (https://www.cochranelibrary.com (accessed on 26 December 2022)). The identification of the relevant literature was conducted by means of key terms and Boolean operators combined in the following search algorithm: ((((breast cancer[Title/Abstract]) AND melatonin[Title/Abstract]) AND English[Language])) NOT REVIEW[Publication Type] AND (Intervention Studies OR intervention OR controlled trial OR “randomized” OR “randomized” OR “random” OR “randomly” OR “placebo” OR “assignment”).

When available, Medical Subject Heading (MESH) terms were used to reinforce the identification of all studies focused on the topic matter of the revision. Moreover, a systematic search was completed with hand searches.

### 2.2. Inclusion and Exclusion Criteria

Reviews were excluded and studies included were restricted to those published in the English language (26 December 2022). Of the results obtained following the aforementioned search strategy, only RCTs that co-administered melatonin in combination with at least one other chemotherapeutic agent to breast cancer patients were considered. Likewise, only studies comparing melatonin administration with a control or placebo group were included. Routes other than the oral administration (e.g., topical, systemic) of melatonin were excluded from the study.

### 2.3. Study Selection

Search results were exported to EndNote (https://www.myendnoteweb.com (accessed on 26 December 2022)), and duplicate publications were automatically or manually removed. Thereafter, eligible articles were selected based on their title, abstract, or full content when required. Studies included RCTs with chemotherapy-treated cohorts and control groups. Due to the low number of RCTs available in the literature, all studies dealing with combined chemotherapy and melatonin regimens were admitted. On the other hand, any disagreement regarding eligibility and/or inclusion was resolved by consensus.

### 2.4. Quality Assessment of Bias of the Selected Studies

To assess the methodological quality of the selected studies, we used the Cochrane collaboration tool (RoB 2) for assessing the risk of bias [45]. For each RCT, several sources to assess the risk of bias were considered. Indeed, RCTs included were assessed for the following domains of bias using the Cochrane tool RoB 2. Domain 1 refers to the randomization process; Domain 2 indicates the risk of deviations from the intended interventions; Domain 3 reflects any missing outcome data; Domain 4 is for outcome measurement; and Domain 5 refers to the selection of the reported result.

The domains for each study were graded as low risk, high risk, or of some concern risk of bias depending on whether available information was adequate, inadequate, or insufficient, respectively. The classification was developed independently by two authors (E.R. and A.R.), aside from the algorithm result of the RoB 2 toll. After grading, with the assistance of RoB 2 toll, the overall quality of each study was considered, and the final scores were discussed by all authors.

The described protocol is fully reproducible and allows for the inclusion of emerging information through newly updated or continually updated systematic reviews, which are also known as living systematic reviews [46].

## 3. Results

### 3.1. Selection and Identification of Relevant Studies

**Figure 1:**
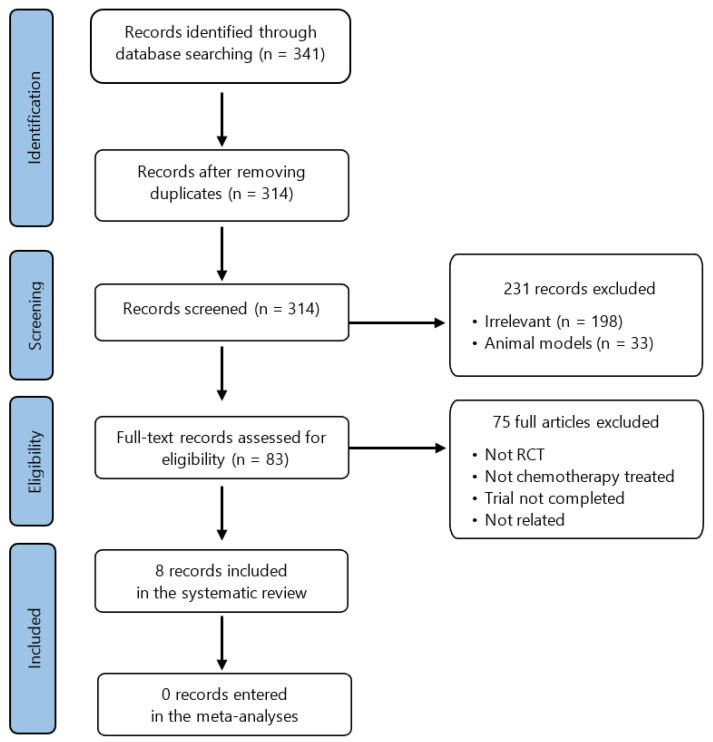
PRISMA flow diagram.

After the algorithmic and manual screenings of the aforementioned databases, 341 records were initially identified, of which 27 duplicates were removed. Of the 314 records reviewed, 198 were considered irrelevant and 33 corresponded to animal studies. In addition, the exclusion criteria applied were non-randomized controlled studies and non-chemotherapy-treated patients. Finally, only eight primary studies of breast cancer patients treated with chemotherapy and melatonin could be considered for the analysis. A detailed flowchart of the selection process is represented in Figure 1.

### 3.2. Characteristics of the Studies

Table 1 summarizes the main features of the included studies. We observed variations concerning doses, study design, and criteria for estimating patient outcomes in the selected RCTs. Overall, five articles included doses from 18–20 mg/day and three from 3–10 mg/day administered from 10 days to 36 months. Three studies included a placebo group, and a comparison was made with a non-melatonin-treated group in five studies, where patients were only treated with chemotherapy. It should be noted that it is highly probable that the variability of patient outcomes was also conditioned by the length of the trials.

The summary of the risk of bias (Table 2) was elaborated using the Cochrane collaboration tool RoB 2. In the case of insufficient information for domain assessment, the study was classified as “no information”. The quality assessment and risk of bias on five different quality domains reflected that none of the selected studies were classified as having a high risk of bias. As can be seen in Table 2, the overall quality of the RCTs considered ranged from low risk to some concerns. Due to the nature of the selected outcomes, no critical concerns were detected in the eight studies.

## 4. Comparative Doses

In the literature, several in vivo studies included melatonin as a co-treatment for breast cancer models. These preclinical investigations pointed out that co-treatment with melatonin would positively influence several clinical outcomes in breast cancer patients. Table 3 summarizes the effects of melatonin co-treatment in animal breast cancer studies. Research in this field showed the therapeutic potential of melatonin to improve the results in treated animals compared with those cancer animals not treated with indoleamine. One of the most frequent observations was the ability of melatonin to reduce tumor size [40,56,57,58,59,60,61,62,63], decrease the risk of metastasis [57,63,64,65,66] and angiogenesis [56,58,61,63,64], and enhance tumor stabilization rates [56,61,67,68,69].

To establish a correlation between melatonin doses in animal studies and RCTs, we calculated the human equivalent doses (HEDs) [70] (Table 3), which are based on the body surface area (BSA) of the animal calculated from body weight and length. The HED calculation is a critical step in ensuring that a drug dose is safe and effective for humans. It helps to avoid potential adverse effects or under-dosing that could compromise the efficacy of a drug. However, it is important to note that the HED has limitations, such as individual variability in metabolism and drug response, which must be taken into account in clinical trials. In this regard, the translation of optimal melatonin doses can be difficult to define because they vary depending on melatonin receptor desensitization, which can result from a variety of mechanisms, including receptor functionality (downregulation, internalization, conformational changes) and/or oscillations in downstream signaling pathways [71]. Additionally, the response of melatonin receptors to high doses of melatonin may depend on the receptor subtype, the treatment schedule (duration and frequency of exposure), and/or the cellular environment [72]. Therefore, although the exogenous administration of melatonin may be beneficial by potentiating antioxidant activity, it may also induce inappropriate responses, such as decreased responsiveness or even pro-oxidant effects [73]. In summary, it is important to carefully consider the dose and duration of melatonin treatment to achieve therapeutic efficacy and minimize possible spurious effects. These estimates are necessary to correctly translate active doses from animal to human studies and understand preclinical results in the context of RCTs. Table 3 also depicts some relevant findings of animal models related, to some extent, to those observed in breast cancer RCTs screened.

The observed outcomes in the selected RCTs are not as positive for melatonin co-administration as reported in preclinical studies. Although, the RCTs with 20 mg/day of melatonin showed very promising results in reducing chemotherapy toxicity, displaying higher partial responses and higher 1-year survival rates. Comparing results with preclinical data, we observed more extensive and encouraging findings as HED doses increased.

When effective animal doses were extrapolated to humans, the HED ranged from 1 to 486 mg per day. The eight clinical trials selected by the PRISMA guidelines tested doses from 3 to 20 mg of melatonin per day. This means that in most animal studies, equivalent doses calculated were significantly greater, even 24-fold greater than those administered to breast cancer patients in RCTs.

## 5. Discussion

Systematic methods based on standardized and explicit protocols make it possible to compile relevant research (primary papers or reports) related to a specific question (a topic of interest) and analyze the information according to objective quality criteria. The backbone of the systematic reviews is schematically reflected in the flow diagram in order to facilitate future reproductions and/or updates of the results and conclusions reached at any time. Thus, the present work is the first systematic review providing a comprehensive analysis of the evidence regarding the effectiveness of melatonin in counteracting chemotherapy-induced toxicity in breast cancer patients. The broad pleiotropy of this indoleamine gives it a presumed pivotal role in many signaling pathways impaired by malignant transformation and, due to its anti-estrogen activity, it is suggested as an ideal candidate for the prevention and/or regulation of the response to treatments. Thus, as far as breast cancer is concerned, diverse recent studies have pointed out that supplemental melatonin can synergistically potentiate drug cytotoxicity. Thus, in vivo and in vitro evidence in breast cancer cells support adjuvant melatonin-sensitized therapy-resistant, primary HER2-positive breast cancer cells for chemotherapy through increased oxidative stress in the endoplasmic reticulum [40]. Similarly, melatonin synergized the effectiveness of several chemotherapy lines by enhancing apoptosis of breast cancer cells [76,77,78,79] or inhibiting the progression of triple-negative breast cancer in a xenograft mouse model through the downregulation of two competing endogenous RNA antagonists of miR-101 [80]. Preclinical results also suggest that melatonin pretreatment prior to radiation sensitizes breast cancer cells to ionizing radiation [81], definitively emphasizing the translational importance of designing melatonin as an adjuvant in radiochemotherapy regimens [82]. Melatonin has also demonstrated proactivity in disrupting the EMT program that directs the implantation of vascular structures, and in VEGF-mediated paracrine regulation of the tumor microenvironment responsible for estrogen production and resistance to anti-angiogenic drugs [83,84,85]. In addition to breast tumor-suppressive capacity by favorably mediating therapy efficacy, supplementation with melatonin restores nocturnal endogenous levels disturbed by chemotherapy, improving sleep quality, circadian cyclicity, and the overall welfare of treated patients. It should be mentioned that in other studies, prescribed melatonin failed to affect breast cancer biomarkers in postmenopausal women with a prior history of breast cancer who had completed active cancer treatment [86], as well as ameliorate insomnia, fatigue, and other detrimental cancer-related symptomatologies [87].

In view of the functional biology of melatonin in breast cancer, the PRISMA methodology allowed the selection of RCTs that addressed this issue and evaluated the protective effects of oral melatonin in breast cancer patients treated with chemotherapy. Additionally, some promising evidence has been reported, such as the finding that topical melatonin counteracts the effects of radiotherapy in breast cancer patients [42]. To date, only eight RCTs have studied melatonin in this regard [47,48,49,50,51,52,53,54,55]. Among them, six tested melatonin at doses ranging from 10 to 20 mg/day and two at 3 mg/day. Overall, they involved 403 breast cancer patients, all of whom had different chemotherapy regimens. These studies showed that compared with placebo or non-treated patients, melatonin co-treatment reduced drug-induced toxicity, promoted higher partial responses, and increased 1-year survival rates. Correspondingly, adjuvant therapy with melatonin should be studied in this context as a way to reduce these side effects and improve the quality of life of breast cancer patients.

Additionally, we first calculated the translational doses of melatonin from the preclinical studies with animals to HED values and subsequently compared them to the doses used in RCTs to interrogate whether doses from experimental studies would be effective for human patients with breast cancer. Of note, the doses used in the reported RCTs have shown melatonin to be effective in reducing chemotherapy-induced toxicity at doses of 10 to 20 mg/day, as well as improving tumor regression rates at doses of 20 mg/day. RCTs with lower dosages of 3 mg/day revealed that melatonin improved sleep quality and fatigue. Mills et al. [88] observed that doses of melatonin ranging from 20–40 mg/day were effective in reducing the risk of cancer. However, it is important to emphasize that more research is needed to determine the optimal translational dose of melatonin in breast cancer patients. The dosages used in studies may not always be directly applicable to human patients, and individual variability in response to melatonin must also be taken into account.

Remarkably, no serious adverse effects were reported in these eight RCTs as a consequence of melatonin co-treatment. Moreover, several chemotherapy-induced toxicities, such as thrombocytopenia, myelosuppression, malaise, asthenia, stomatitis and neuropathy, leukopenia, and anemia or cardiac complications, were less common in melatonin-treated patients [47,48,49,50,53,54]. Therefore, we can conclude that melatonin does not appear to have safety concerns, but rather counteracts chemotherapy-related adverse effects. In this line, melatonin’s safety has been extensively reviewed in the literature [89,90,91].

Several limitations should be considered when reaching conclusions from the selected RCTs for this review. These limitations include the small number of RCTs performed that were available in the literature. Concerning the methodology of the RCTs, the lack of blinding the participants (medical staff and patients) increases the risk of incurring biases. However, objective assessments of patient outcomes would not likely be altered by insufficient blinding. Nonetheless, the heterogeneity of chemotherapy cycles and outcomes limited general conclusions because they made direct comparisons or meta-analyses on the efficacy of melatonin as a coadjuvant in breast cancer therapy impossible.

One of the limiting factors that prevent the direct extrapolation of the results from animal studies to humans is the fact that experimental animals (mainly rodents; mice and rats) tend to be nocturnal, whereas humans are diurnal. Intriguingly, circadian rhythm patterns can affect cancer development and progression [92]. Nocturnal animals have different metabolic rates than daytime animals, which may affect the absorption, distribution, and elimination of drugs and other active substances used in cancer treatment [93]. Furthermore, nocturnal animals exhibit different immune responses than humans and also different genetic backgrounds than humans, which may affect disease susceptibility, gene expression, drug metabolism, and other biological processes [94]. Therefore, while animal studies can provide valuable insights into cellular biochemistry and how it is affected by pharmacological and other interventions, it is important to keep in mind the limitations of extrapolating findings from research models to humans.

Melatonin is used as part of circadian-based regimens to maximize drug efficacy and tolerability (oncochronotherapies) by timing the delivery to match the body’s circadian rhythms. Thus, it has been suggested that administering melatonin at the appropriate phase of the circadian cycle may enhance its anti-tumor activity and reduce the side effects of chemotherapy and radiation therapy [95]. In this regard, while the optimal dosage of melatonin is an important consideration in cancer research, the timing of administration is also crucial to maximize its anti-cancer and other beneficial effects. The need for researchers to carefully consider the chronodependence of melatonin administration in their investigations and take into account the circadian rhythms in animal studies and clinical trials must be emphasized.

A great number of in vivo and in vitro reports on the efficacy of melatonin and its mechanisms of action against cancer models can be found in the literature [96,97], including its ability to regulate cell proliferation, apoptosis, and immune system function. Nevertheless, despite the promising actions reported in this systematic review, only a few RCT-reported studies have been conducted to date. Although the available clinical research is scarce, some RCTs have treated post-operative breast cancer patients with no additional chemotherapy and compared them with the placebo group, finding that melatonin led to better sleep quality and reduced depressive symptoms [98,99,100]. However, more clinical studies are needed to determine the optimal dose, timing, and route of administration of melatonin in cancer-treated patients.

## 6. Conclusions

Despite numerous animal-based studies available in the literature, only a few clinical trials confirming the benefits of melatonin co-treatment for breast cancer patients have been developed. The eight RCTs included in this review provide evidence for the safety and positive outcomes of melatonin as a therapeutic agent. However, these results are not sufficient evidence to conclude that clinical co-administration of melatonin may be effective due to the small number of trials carried out and the heterogeneity of treatment conditions and, consequently, of the outcomes reported. Furthermore, the doses to be administered in future RCTs should be reconsidered since we demonstrated that the direct extrapolation of effective doses in animal models to humans leads to doses that are likely too low to significantly influence tumor regression, metastasis, and/or angiogenesis. Therefore, more RCTs with more accurate melatonin doses are needed to confirm whether this indoleamine can generate synergies with chemotherapy that improve outcomes in breast cancer patients. In this regard, the potential benefits of melatonin co-treatment in improving the quality of life for cancer patients make it a promising avenue for future research.

## Figures and Tables

**Table 1:** Characteristics of the included trials with breast cancer patients.

Study	Country	Sample Size	Median Age	Mel mg/day	CT	Period	Improved Outcome
[47]	Italy	40	65–66	20	tamoxifen	6–7 months	CT-induced toxicity Partial response 1-year survival rate
[48]	Italy	31	59	20	mitoxantrone	5–7 months	CT-induced toxicity
[49]	Italy	77	59	20	doxorubicin paclitaxel mitoxantrone	36–12 months	Tumor regression rate 1-year survival rate CT-induced toxicity ^a^
[50,51]	Brazil	36	54	20	CT	10 days	Pain perception
							Cognitive function, sleep quality, and depressive symptoms.
[52]	Russia	54	68	3	toremifene	4 months	Sleep quality
[53]	Russia	53	56	3	NACT	6 months	CT-induced fatigue and sleep quality
[54]	Iran	78	50–46	18	CT and RT	25 weeks	CT-induced fatigue
[55]	Iran	34	51–52	10	paclitaxel	12 weeks	Neuropathic chronic pain

CT = chemotherapy; RT = radiotherapy; NACT = anthracycline and taxan-containing neoadjuvant chemotherapy. ^a^ Results were not significant in mitoxantrone-treated patients.

**Table 2:** Quality assessment of randomized clinical trials according to the revised guidelines of the Cochrane collaboration tool RoB 2 for the risk of bias.

Risk Domain/Reference	[47]	[48]	[49]	[50]	[52]	[53]	[54]	[55]
Randomization process	+	+	+	+	+	+	+	+
Deviations from intended interventions	+	+	+	+	!	!	+	+
Missing outcome data	+	+	+	+	+	+	+	+
Measurement of the outcome	+	+	+	+	+	+	+	+
Selection of the reported result	!	!	!	+	+	+	+	+
Overall	!	!	!	+	+	!	+	+

Green (+) = low risk; yellow (!) = some concerns.

**Table 3:** Therapeutic results of preclinical studies and a comparison of the active doses in animal studies and RCTs (the HED extrapolation dose for human adults).

Animal Model	Ref.	Findings	Melatonin Daily Dose	Administration Period	Daily HED for a 60 kg Adult
Mouse	[67]	Enhances stabilization rates	0.2 mg/kg	3 weeks	1 mg
	[56]	Reduces tumor size, cell growth, and angiogenesis	2 mg/kg	2 weeks	10 mg
	[74]	Diminishes angiogenesis and cell survival	2 mg/kg	2 weeks	
	[75]	Inhibits mammary carcinogenesis	2.5–3.1 mg/kg	9 months	12–15 mg
	[57]	Reduces tumor size and formation and metastasis Longer survival	5 mg/kg	10 weeks	24 mg
	[65]	Diminishes cell growth, survival, and metastasis	20 mg/kg	4 weeks	97 mg
	[64]	Diminishes metastasis and angiogenesis	30 mg/kg	27 days	146 mg
	[58]	Reduces tumor size and angiogenesis	40 mg/kg	3 weeks	195 mg
	[59]	Reduces tumor size	40 mg/Kg	3 weeks	
	[60]	Reduces tumor size	40 mg/kg	3 weeks	
	[40]	Reduces tumor size and inhibits cell growth	50 mg/kg	27 days	243 mg
	[66]	Diminishes metastasis	100 mg/kg	2/5 weeks	486 mg
Rat	[68]	Antiproliferative and immunomodulatory effects	1.2 mg/kg	16 weeks	12 mg
	[61]	Decreases tumor frequency and tumor size and extends tumor latency. Antiangiogenic, bone metabolism	2.1 mg/kg	15 weeks	20mg
	[69]	Decreases tumor frequency and extends latency	2.4 mg/kg	15 weeks	23 mg
	[62]	Reduces tumor size	2.5 mg/kg	120 days	24 mg
	[63]	Reduces tumor size Diminishes metastasis and angiogenesis	10 mg/kg	2 weeks	97 mg

## Data Availability

Not applicable.
